# Gender Differences in Fat Distribution and Inflammatory Markers among Arabs

**DOI:** 10.1155/2013/497324

**Published:** 2013-10-21

**Authors:** Abdulaziz Farooq, Wade L. Knez, Kelly Knez, Asma Al-Noaimi, Justin Grantham, Vidya Mohamed-Ali

**Affiliations:** ^1^Aspetar, Qatar Orthopaedic and Sports Medicine Hospital, P.O. Box 29222, Doha, Qatar; ^2^Supreme Council of Health, P.O. Box 7744, Doha, Qatar; ^3^Aspire Zone Foundation, P.O. Box 93097, Doha, Qatar; ^4^University College London, 5 University Street, London WC1E 6JF, UK; ^5^Life Sciences Research Division, Anti-Doping Lab Qatar, P.O. Box 27775, Doha, Qatar

## Abstract

Recent studies from the Gulf region suggest that compared to men, women have a greater risk of developing metabolic syndrome (MeS). *Objective*. To investigate gender differences in body composition, adipokines, inflammatory markers, and aerobic fitness in a cohort of healthy Qatari adults. *Participants*. Healthy Qatari (*n* = 58) were matched for age, gender, and body mass index. *Methods*. Body composition and regional fat distribution were determined by dual-energy X-ray absorptiometry and computerized tomography. Laboratory assessments included serum levels of fasting glucose, insulin, lipid profile analysis, adipokines, and inflammatory markers. Subjects were also evaluated for aerobic fitness. *Results*. Women had more adipose tissue in the total abdominal (*P* = 0.04) and abdominal subcutaneous (*P* = 0.07) regions compared to men. Waist circumference and indices of insulin sensitivity were similar; however, women had a more favourable lipid profile than men. Serum adiponectin and leptin levels were significantly higher in women, whereas inflammatory profiles were not different between men and women. Aerobic fitness was lower in women and was associated with abdominal fat accumulation. *Conclusion*. In premenopausal women, higher levels of adiponectin may support maintenance of insulin sensitivity and normolipidemia despite greater adiposity. However, poor aerobic fitness combined with abdominal fat accumulation may explain their greater future risk of MeS compared with men.

## 1. Introduction

The metabolic syndrome (MeS) is defined as a cluster of interrelated metabolic abnormalities that doubles the risk of type 2 diabetes mellitus and cardiovascular disease (CVD) [1]. The major features of MeS are insulin resistance, central obesity, hypertension, and dyslipidaemia. This condition is often associated with suppression of adiponectin and elevation of leptin and various inflammatory markers (e.g., interleukin-6 [IL-6], monocyte chemoattractant protein-1 [MCP-1], C-reactive protein [CRP], and regulated on activation, normal T cell expressed and secreted [RANTES]) [2, 3] that may play a causal role in insulin resistance. Also, insufficient physical activity accompanied with increased or inappropriate fat accumulation [4, 5] may potentially increase the risk of CVD [6].

The prevalence of MeS in Gulf Cooperation Council (GCC) countries is ranked amongst the highest in the world [7]. Furthermore, the risk of MeS is higher amongst Arab women (13–55%) compared to Arab men; it is 18% higher in women in Oman [8, 9], 55% in Qatar [10], 22% in United Arab Emirates [11], and 13% in Saudi Arabia [12]. On the other hand, data from Caucasian populations either point to men and women being equally at risk of developing MeS [13–15] or to men being more predisposed to developing MeS than women [16–21]. This difference in MeS risk is thought to be, at least partly, explained by men having a greater propensity for abdominal obesity compared to premenopausal women. It is unclear as to what factors confer the reported higher risk of developing MeS in Arab women compared to men. Gender differences in fat accumulation and/or the secretory function of adipose tissue may explain some of this disparity. Poor aerobic fitness, in addition to low grade inflammation, contributes to the development of insulin resistance [22] and is often a better predictor for CVD risk factors than self-reported physical activity [23]. Furthermore, muscular strength and aerobic fitness combined or independently are inversely related to MeS [24]. Therefore, this study investigated gender differences in body composition, systemic levels of adipokines and inflammatory markers, and aerobic fitness in a cohort of healthy Qatari adults matched for age and body mass index (BMI).

## 2. Methods

### 2.1. Study Subjects

A matched case-control study was conducted at Aspetar, Qatar Orthopaedic and Sports Medicine Hospital between February 2009 and December 2009. This study was approved by the Institutional Research Ethics Committee and all subjects provided written consent prior to participation. Healthy Qatari men (*n* = 29) and women (*n* = 29) were matched for age and BMI. Subjects with diabetes mellitus, those who were pregnant or postmenopausal, or receiving medical treatment for any chronic disease were excluded from the study. After a 10-hour overnight fast, subjects underwent a detailed clinical assessment, including body composition, fat distribution, anthropometry measurements, and blood pressure. Blood was drawn for haematological assessment and measurement of various metabolic and inflammatory markers. Subjects also underwent a series of tests for aerobic fitness and indices of muscular strength.

### 2.2. Anthropomorphic Assessment

Measurements included height, weight, and waist circumference. Height was measured to the nearest 0.1 cm (Seca 242, Germany), and weight was measured to the nearest 0.1 kg using a portable stadiometer (Detecto, USA). Both height and weight measurements were recorded without shoes and in light-weight clothing. Waist circumference was measured to the nearest 0.1 cm at the smallest girth horizontally around the trunk underneath the subject's clothing. Central obesity was defined as >80 cm for women and >90 cm for men [25]. Two blood pressure readings were taken 5 minutes apart with the subject at rest in a relaxed sitting position. The average systolic and diastolic blood pressures were calculated and used in subsequent analyses.

### 2.3. Body Composition Assessment

DXA (GE Medical System Lunar, Madison, Wisconsin, USA) using the enCORE software (version 12.10) was used to quantify fat mass (g), tissue (g), lean mass (g) and percentage of body fat. CT scans were performed to obtain 5 axial images of each of the following regions: heart, liver, abdomen, and midthigh. For the abdominal region, cross-sectional axial images of the L4-L5 vertebral disc space were obtained. Omental adipose tissue was differentiated from subcutaneous adipose tissue by manual drawing, and subcutaneous adipose tissue was further classified as superficial and deep. Two cross-sectional axial images of the left and right thigh at the femoral midpoint region were obtained. Intramuscular adipose tissue and subcutaneous adipose tissue in the thigh were distinguished by manual drawing using the right thigh image. An upper limit of −30 Hounsfield units (HU) and a lower limit of −190 HU were used to differentiate adipose tissue from other tissue types on the CT images. All volumetric analyses were performed by an experienced radiologist using the Somaris/5 Syngo CT2006A system (Siemens, Germany). All subjects wore standard hospital gowns during the DEXA and CT scan procedures.

### 2.4. Haematological Assessment

A complete blood count was performed, and serum levels of iron, ferritin, iron-binding capacity, transferrin, and lipids, including high-density lipoprotein (HDL), low-density lipoprotein (LDL), total cholesterol, and triglycerides, determined by the Pathology Laboratory at Aspetar. Fasting plasma glucose (Beckman, CA, USA) insulin (Mercodia, Uppsala, Sweden) was also assessed, and the HOMA-IR (homeostasis model of assessment-insulin resistance) was calculated using the following formula: (fasting insulin in mIU/L × fasting glucose in mmol/L)/22.5 [26]. Fasting serum levels of CRP, adiponectin, leptin, RANTES, MCP-1, and IL-6 were measured using human 2-site ELISAs (R&D Systems, Oxon, UK). IL-6 concentrations were assayed with the high sensitivity ELISA with a limit of detection of 0.09 pg/mL. All inter- and intraassay CVs were less than 10%.

### 2.5. Fitness and Strength Assessment

Aerobic fitness was assessed using the Bruce treadmill test. Starting speed was set to 2.74 km/hr, and the intensity (speed and incline) was increased by 2% every 3 minutes until volitional exhaustion. Peak heart rate (HR), percentage of predicted maximal HR, test duration, and peak oxygen uptake were measured. Subjects were also evaluated for hand grip strength and leg strength. The hand grip strength was measured with the Lode dynamometer (Lode BV, Groningen, The Netherlands). Leg strength was assessed by recording isokinetic knee flexion and extension concentrically (at 30°/second and 120°/second) and isometric extension at 90° on the dominant leg (Biodex 3.0 system, Version 3.4, Shirley, NY, USA). 

### 2.6. Statistical Analysis

Data were analysed using the SPSS (Statistical Package for the Social Sciences, version 15.0) software. Descriptive statistics included mean ± SD for normally distributed data and median (interquartile range, IQR) for skewed data. An independent sample *t*-test was used to compare continuous data between two groups and the nonparametric equivalent Mann-Whitney *U* test was used when appropriate. Log transformations were applied to normalize the distribution of adipokines and cytokines before computing Spearman's correlation coefficients against metabolic markers. Data from this study population was compared to the published age- and gender-specific percentiles for aerobic fitness from the Aerobic Center Longitudinal Study (ACLS) [27]. *P* values less than 0.05 were considered to be statistically significant.

## 3. Results

A total of 29 men and 29 women matched for age and BMI were included in the study, with a mean age of 33.4 ± 10.3 years (Table 1). Despite the similarity in BMI and waist circumference, body fat percentage was higher in women (women: 43.4 ± 6.3% versus men: 32.7 ± 8.8%, *P* < 0.01). Women had significantly lower levels of triglycerides (*P* < 0.01) and LDL (*P* = 0.05) and higher levels of HDL (*P* < 0.01), compared to men. Women had lower values for various iron indices compared with men: serum iron (*P* = 0.05), serum ferritin (*P* < 0.01), and haemoglobin level (*P* < 0.01). Indices of insulin sensitivity and glucose handling (insulin, HOMA-IR, fasting glucose, and HbA1c levels) were similar in men and women.

Total fat in the abdominal region was higher in women compared to men, especially in the deep subcutaneous abdominal region (Table 2). There were no differences in adipose tissue in the omental abdominal or liver regions. Adiposity was also significantly higher in women than men in the heart intra- (*P* = 0.04) and overall heart (*P* < 0.01) regions, as well as in the thigh region (*P* < 0.01).

**Figure 1:**
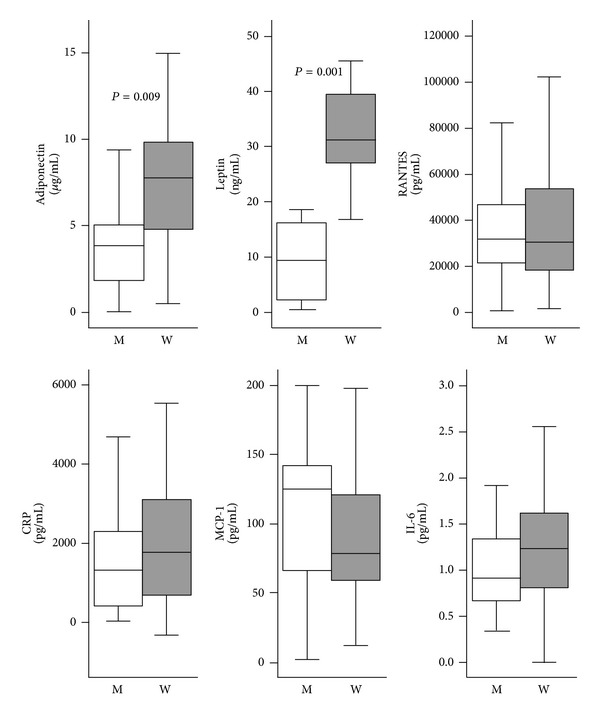
Serum levels of adipokines and inflammatory markers by gender. Data are expressed as Median (IQR) with different scales for each adipokines/cytokines. Only significant *P* values (Mann-Whitney *P*) are shown. M: Men; W: Women; CRP: C-reactive protein; RANTES: regulated on activation, normal T cell expressed and secreted; MCP-1: monocyte chemotactic protein-1; IL-6: interleukin-6.

Gender differences in the correlations between serum metabolic markers, adipokines/cytokines, and VO_2_ Max were investigated (Table 3). In men, adiponectin and RANTES were not associated with any of the metabolic markers studied, whereas in women, adiponectin was negatively correlated with triglycerides (*r* = −0.45, *P* = 0.02) and insulin (*r* = −0.39, *P* = 0.05). In both men and women, CRP was negatively correlated with aerobic fitness (*r* = −0.43, *P* = 0.05 and *r* = −0.54, *P* < 0.01 resp.). IL-6, leptin, and CRP were strongly correlated with waist circumference and systolic BP only in men (Table 3). Serum adiponectin and leptin levels were significantly higher in women than men, whereas RANTES, CRP, MCP-1, and IL-6 were not different (Figure 1).

Tests of aerobic fitness showed that both men and women reached at least 95% of their age-predicted maximum HR (Table 4). On average, women ran on the treadmill for almost 7 minutes, whereas men completed 9.5 minutes (*P* < 0.01). Calculated aerobic fitness (VO_2_ Max) was markedly higher in men than women (*P* < 0.01) as were all indices of leg and hand grip strength (*P* < 0.01). Using multiple regression analysis, we found that gender was the strongest determinant for poor aerobic fitness (−7.5, 95% CI: −10.3 to −4.8) followed by waist circumference (−0.22, 95% CI: −0.34 to −0.11), after adjusting for BP.

## 4. Discussion

Reports suggest that prevalence of coronary heart disease is higher in men and postmenopausal women. [17, 28, 29]. However, in the Arab population, coronary heart disease-associated risk of morbidity and mortality are elevated even in younger women compared to other ethnic groups [30, 31]. This study sought to clarify the gender differences in fat distribution, serum markers of metabolism and inflammation, and measures of aerobic fitness in an age- and BMI-matched population of healthy Qatari men and women.

Central fat distribution has been associated with haemostatic and inflammatory markers of MeS [32]. The relatively young premenopausal women in this study had significantly greater fat content in the heart, abdominal, and thigh regions compared with age-matched men (Table 2). However, this fat distribution pattern was also accompanied by a favourable lipid profile (high HDL and low LDL) and elevated adiponectin and leptin in women, compared with men. Subcutaneous fat alone is responsible for 80% of leptin production in the body [3], and thus leptin levels appear to be a good marker for this type of fat deposition in women. The positive correlation of femoral fat with adiponectin (*r* = 0.44, *P* = 0.01) in the present study (Table 3) is consistent with previous findings that gluteofemoral deposition of fat is cardioprotective [33].

Adiponectin was negatively correlated with HOMA-IR (*r* = −0.38, *P* = 0.056) and serum triglycerides (*r* = −0.46, *P* = 0.02) in women, but these relationships were surprisingly absent in men (HOMA-IR; *r* = 0.08, *P* = 0.72 and TG; *r* = 0.04, *P* = 0.84). Adiponectin acts as an endogenous insulin sensitizer, both directly on muscle cells and indirectly through insulin [34], and circulating concentrations of this adipokine are strongly and positively correlated with HDL concentration and negatively correlated with triglyceride levels [35]. While adiponectin may be related to the favourable lipid profile seen in the women in this study, it does not appear to be anti-inflammatory, as both women and men had comparable levels of IL-6, MCP-1, CRP, and RANTES (Figure 1).

The comparable levels of circulating adipokines/cytokines may reflect similar levels of omental adipose tissue amongst the study subjects. Adipose tissue-derived IL-6 and MCP-1 account for approximately 15%–30% of systemic levels of these cytokines in obese individuals [3]. CRP plays a role in inflammation and insulin resistance [3, 36] and may be derived from adipose tissue or elevated as a consequence of higher IL-6 and MCP-1 levels. Adipose tissue also releases RANTES, another putative mediator of impaired glucose tolerance and type 2 diabetes [37].

**Figure 2:**
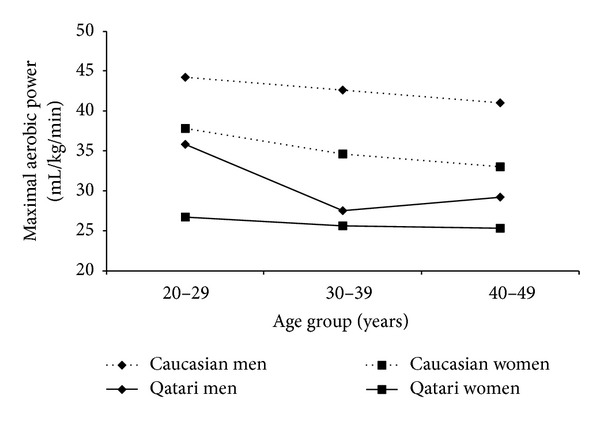
Aerobic fitness in Qatari men and women in the current study, compared to the reported 50th percentile of aerobic fitness in a Caucasian population [27].

Among patients with elevated risk of type 2 diabetes, physical inactivity can aggravate low-grade inflammation [38]. Both men and women in the present study had extremely low levels of aerobic fitness. VO_2_ Max for half of the men (44.8%) and the majority of women (87.5%) was below the 20th percentile reported by ACLS for a Caucasian population [27] (Figure 2). However, for measures of muscular strength, approximately 87.0% of men and 75.9% of women were above the 75th percentile of values reported by ACLS [27].

## 5. Conclusion

Despite higher levels of abdominal body fat compared with Arab men, premenopausal Arab women have elevated serum adiponectin that may maintain normolipidemia and insulin sensitivity. The accumulation of omental adipose tissue, combined with extremely low aerobic fitness, in Arab women may abrogate the protective effect of adiponectin, leading to a greater risk of developing MeS. Further research is needed to understand the gender-specific causal factors that increase the risk of MeS among Arab women.

## Figures and Tables

**Table 1:** Anthropometry, body composition, lipid profile, iron indices, insulin sensitivity, and glucose handling in men and women.

Variable	Men *n* = 29	Women *n* = 29	*P* value
Age (years)	32.6 ± 10.7	34.2 ± 10.1	0.55
BMI (kg/m^2^)	27.9 ± 5.9	28.3 ± 6.1	0.79
Waist circumference (cm)	95.4 ± 17.4	90.1 ± 11.3	0.19
Body fat (%)	32.7 ± 8.8	43.4 ± 6.3	<0.01
Systolic BP (mm Hg)	127.6 ± 11.4	126.3 ± 16.8	0.73
Diastolic BP (mm Hg)	79.2 ± 9.9	77.8 ± 9.9	0.58
Lipid profile			
Total cholesterol (mmol/L)	5.1 ± 1.1	4.7 ± 0.7	0.19
Triglycerides (mmol/L)	1.2 ± 0.5	0.8 ± 0.4	0.01
HDL (mmol/L)	1.2 ± 0.2	1.4 ± 0.3	0.01
LDL (mmol/L)	3.2 ± 0.9	2.8 ± 0.7	0.06
Iron indices			
Haemoglobin (g/dL)	14.8 ± 0.8	12.3 ± 0.7	<0.01
Serum iron (*μ*g/dL)	15.2 ± 4.0	10.8 ± 5.7	0.05
Serum ferritin (*μ*g/dL)	105.9 ± 37.0	23 ± 20.1	<0.01
Iron-binding capacity (*µ*mol/L)	56.9 ± 5.9	62.9 ± 9.6	0.10
Urea nitrogen (mmol/L)	5.0 ± 1.4	3.2 ± 0.7	<0.01
Creatinine (mmol/L)	78.4 ± 17.3	55.8 ± 6.7	<0.01
Insulin sensitivity and glucose handling			
Fasting glucose (mmol/L)	5.0 ± 0.5	5.0 ± 0.6	0.77
Insulin (*μ*IU/mL)	4.23 (3.59–6.39)	6.22 (3.82–10.7)	0.12
HOMA-IR	1.07 (0.77–1.31)	1.44 (0.82–2.24)	0.19
HbA1c (%)	5.6 ± 0.3	5.6 ± 0.4	0.66

Data shown as mean ± SD or median (interquartile range).

BMI: body mass index; BP: blood pressure; HDL: high-density lipoprotein; LDL: low-density lipoprotein; HOMA-IR: homeostasis model of assessment-insulin resistance.

**Table 2:** Regional fat characteristics measured by CT scan.

Variable	Men *n* = 19	Women *n* = 16	*P* value
Heart—total	94.0 ± 68.0	155.0 ± 46.0	<0.01
Heart—intra	19.0 ± 12.0	23.0 ± 7.0	0.04
Liver—total	93.9 ± 63.5	122.3 ± 47.3	0.09
Liver—intra	31.9 ± 20.6	29.9 ± 11.7	0.86
Abdominal—total	187.3 ± 113.3	247.1 ± 65.6	0.04
Abdominal—omental	50.7 ± 27.8	57.8 ± 22.3	0.18
Abdominal—SC	136.6 ± 89.3	176.8 ± 68.8	0.07
Abdominal—SC, super	135.8 ± 86	140.5 ± 35.3	0.33
Abdominal—SC, deep	20.3 ± 19.4	42.4 ± 24.9	0.04
Thigh—total	52.2 ± 31.8	95.5 ± 32.1	<0.01
Thigh—intra	3.2 ± 2.0	6.6 ± 3.6	<0.01
Thigh—muscle	74.0 ± 11.2	54.1 ± 7.7	<0.01

Data shown as mean ± SD.

SC: subcutaneous; super: superficial.

**Table 3:** Correlation between metabolic markers, and serum adipokines and inflammatory markers in men and women.

Metabolic marker	Adiponectin *μ*g/mL	CRP pg/mL	RANTES pg/mL	MCP-1 pg/mL	Leptin ng/mL	IL-6 pg/mL
Men						
Total cholesterol (mmol/L)	0.03	0.25	−0.05	0.14	0.21	0.08
Triglycerides (mmol/L)	0.04	0.07	−0.21	0.46*	0.35	0.00
HDL (mmol/L)	0.23	0.04	−0.06	−0.08	−0.09	−0.01
LDL (mmol/L)	−0.04	0.29	−0.09	0.15	0.17	0.13
Waist circumference (cm)	−0.07	0.64**	−0.06	0.25	0.86**	0.76**
Systolic BP (mm Hg)	0.00	0.63**	−0.21	0.10	0.47*	0.61**
Diastolic BP (mm Hg)	0.05	0.39	−0.21	0.22	0.34	0.48*
Insulin (*μ*IU/mL)	0.05	0.46	0.12	−0.19	0.65**	0.35
Fasting glucose (mmol)	−0.09	0.34	0.18	0.25	0.45	0.31
HOMA-IR	0.08	0.41	0.11	−0.18	0.65**	0.35
VO_2_ Max (mL/kg/min)	0.22	−0.43*	−0.14	−0.03	−0.40	−0.51*
Women						
Total cholesterol (mmol/L)	−0.01	0.15	0.20	0.15	0.21	0.25
Triglyceride (mmol/L)	−0.45*	0.27	0.22	−0.21	0.33	0.26
HDL (mmol/L)	0.28	−0.06	0.01	0.11	−0.01	−0.25
LDL (mmol/L)	−0.07	0.19	0.31	0.10	0.25	0.41*
Waist circumference (cm)	−0.10	0.01	0.14	0.25	0.07	0.53**
Systolic BP (mm Hg)	0.32	0.14	0.24	0.21	0.08	0.17
Diastolic BP (mm Hg)	−0.04	0.13	0.19	0.55*	0.11	0.46*
Insulin (*μ*IU/mL)	−0.39*	0.27	0.28	−0.44	0.50	0.23
Fasting glucose (mmol)	−0.32	0.25	0.39*	−0.18	0.14	0.22
HOMA-IR	−0.38	0.27	0.32	−0.39	0.47	0.27
VO_2_ Max (mL/kg/min)	0.17	−0.54**	0.03	−0.38	−0.34	−0.11

Data reported as Spearman correlation coefficients. Bold font indicates gender-specific correlations. *P < 0.05; **P < 0.01.

CRP: C-reactive protein; RANTES: regulated on activation, normal T cell expressed and secreted; MCP-1: monocyte chemotactic protein-1; IL-6: interleukin-6; HDL: high-density lipoprotein; LDL: low-density lipoprotein; BP: blood pressure; HOMA-IR: homeostasis model of assessment-insulin resistance; VO_2_ Max: maximal oxygen uptake.

**Table 4:** Aerobic fitness and indices of strength.

Variable	Men *n* = 29	Women *n* = 29	*P* value
Peak HR (bpm)	177.6 ± 17.5	177.0 ± 16.8	0.90
Percentage of predicted maximal HR (%)	94.9 ± 8.1	95.1 ± 9.0	0.95
Bruce treadmill test duration (min)	9.5 ± 1.8	6.8 ± 0.7	<0.01
VO_2_ Max (mL/kg/min)	32.5 ± 7.1	26.0 ± 3.2	<0.01
Max handgrip (N)	396.1 ± 88.8	256.3 ± 41.4	<0.01
Knee isometric peak (Nm)	256.8 ± 58.7	155.3 ± 41.8	<0.01
Knee isokinetic 30 degree peak (Nm)	194.2 ± 48.6	121.8 ± 28.3	<0.01
Knee isokinetic 120 degree peak (Nm)	140.6 ± 32.2	82.9 ± 19.3	<0.01

Data shown as mean ± SD.

HR: heart rate; VO_2_ Max: maximal oxygen uptake.
